# Knee Flexor Eccentric Strength, Hamstring Muscle Volume and Sprinting in Elite Professional Soccer Players with a Prior Strained Hamstring

**DOI:** 10.3390/biology11010069

**Published:** 2022-01-03

**Authors:** Alberto Mendez-Villanueva, Francisco Javier Nuñez, Jose Luis Lazaro-Ramirez, Pablo Rodriguez-Sanchez, Marc Guitart, Gil Rodas, Imanol Martin-Garetxana, Josean Lekue, Valter Di Salvo, Luis Suarez-Arrones

**Affiliations:** 1 Qatar Football Association, Doha 999043, Qatar; amendezvillanueva@yahoo.com; 2 Department of Sport and Informatics, Section of Physical Education and Sport, Pablo de Olavide University, 41013 Sevilla, Spain; fjnunsan@upo.es (F.J.N.); jose.lazaro2327@gmail.com (J.L.L.-R.); pablonf7@hotmail.com (P.R.-S.); 3 Medical and Performance Department, Futbol Club Barcelona, 08028 Barcelona, Spain; marc.guitart@fcbarcelona.cat (M.G.); gil.rodas@fcbarcelona.cat (G.R.); 4 Medical Services, Athletic Club, 48196 Lezama, Spain; i.martin@athletic-club.eus (I.M.-G.); j.lekue@athletic-club.eus (J.L.); 5 Department of Physiology, Faculty of Medicine and Nursing, University of the Basque Country (UPV/EHU), 48940 Leioa, Spain; 6 Department of Movement, Human and Health Sciences, University of Rome “Foro Italico”, 00135 Rome, Italy; valter.disalvo@aspire.qa; 7 Performance Department, FC Basel 1893, 4057 Basel, Switzerland

**Keywords:** hamstring injury strain, Nordic hamstring exercise, muscle morphology, running, football

## Abstract

**Simple Summary:**

Given the link between a prior hamstring injury and the increased risk of suffering a subsequent injury in professional soccer, an understanding of the features displayed by previously injured players is important. The aim of the present study was to investigate if players with a history of hamstring injury exhibit bilateral deficits in knee flexor eccentric strength, hamstring muscle volume, and sprinting performance. The eccentric knee flexor strength was greater in the previously injured limbs compared to both the contralateral uninjured limbs in the previously injured group and the uninjured limbs in the previously uninjured players. Previously injured limbs showed possibly larger short heads of the biceps femoris and likely semitendinosus muscle volumes. Sprinting performances in the 5-m were possibly worse in players with a history of hamstring muscle strain injury compared to the previously uninjured players.

**Abstract:**

The aim was to determine if players with a prior hamstring strain injury (HSI) exhibit bilateral deficits in knee flexor eccentric strength and hamstring muscle volume and differences in sprinting performance compared with players without a history of HSIs. Forty-six male professional soccer players participated in this study. Eccentric knee flexor strength, hamstring muscle volume (MRI), and a 20-m running sprint test (5- and 10-m split time) were assessed at the start of the preseason. Eccentric knee strength of the previously injured limbs of injured players was greater (ES: 1.18–1.36) than the uninjured limbs in uninjured players. Previously injured limbs showed possibly larger biceps femoris short heads (BFSh) and likely semitendinosus (ST) muscle volumes than the contralateral uninjured limbs among the injured players (ES: 0.36) and the limbs of the uninjured players (ES: 0.56), respectively. Players who had experienced a previous HSI were possibly slower in the 5-m (small ES: 0.46), while unclear differences were found in both the 10-m and 20-m times. Players with a prior HSI displayed greater eccentric knee flexor strength, possibly relatively hypertrophied ST and BFSh muscles, and possibly reduced 5-m sprinting performances than previously uninjured players. This can have implication for the design of secondary hamstring muscle injury prevention strategies.

## 1. Introduction

Hamstring strain is the most common injury in soccer players and are related to substantial time loss and high economic costs for the players and soccer clubs [1,2]. Consequently, adequate prevention and rehabilitation strategies are very important within this cohort. However, despite concentrated prevention and rehabilitation efforts, injury and reinjury rates have continued to be consistently high in professional European soccer in the last fifteen years [3], accounting for more than one-third of all time-loss injuries and causing more than 25% of the total injury absenteeism in European professional soccer teams [4].

A previous hamstring strain injury (HSI) has been recognised as the most prominent risk factor to sustain a hamstring injury in professional soccer [5]. Although previous HSI is a nonmodifiable risk factor, targeted intervention measures can be implemented with those players. Importantly, prospective studies in soccer players have identified an inter-relationship between the nonmodifiable risk factor of previous hamstring injury history with modifiable factors such as knee flexor eccentric strength [6,7]. For example, Timmins et al. (2016) indicated that the risk of a subsequent HSI in soccer players with a history of HSI was lower when coupled with absolute high levels of eccentric knee flexor strength [6]. Similarly, low knee flexor eccentric strength was able to predict the recurrence of HSI within a cohort of amateur soccer players [7]. Accordingly, eccentric strength abilities might be considered one of the key factors for secondary prevention. Indeed, football teams from around the world deemed eccentric exercises and, specifically, hamstring eccentric exercises, as the most important to prevent noncontact injuries [5]. However, despite this evidence and perceived importance in professional football, based on the current research literature, hamstring eccentric strength was only given a weakly graded recommendation for the use in the practical setting [5].

Some retrospective and prospective reports have identified persistent atrophy (i.e., loss in muscle volume) in previously injured hamstrings in different athletic populations [8,9]. Interestingly, some reports have shown that the reduction in muscle volume appeared to be circumscribed to the injured hamstring muscle belly with no difference in agonist or antagonist muscle volume [8]. Considering that HSI in soccer most commonly involves the biceps femoris long head muscle belly, accounting for approximately 60–85% of all hamstring injuries [10,11,12,13], it might be possible that some atrophy occurs near this region. Indeed, a significant reduction in biceps femoris long head volume was identified in over 50% of subjects with a prior HSI despite having returned to athletic competition [9]. Moreover, in addition to biceps femoris long head muscle atrophy [9], shorter muscle fascicle length [14] and hypertrophy of the biceps femoris short head [9,15] were detected in previously injured hamstring muscles in comparison with the uninjured limb. However, to date, it is unknown if a previously strained hamstring displays morphological (e.g., muscle volume) differences compared to an uninjured hamstring in professional soccer players.

A significant number of HSI in soccer occur during rapid accelerations and sprinting movements [2,16]. Hamstring muscles are active during the stance and swing phases of sprinting [17] and are assumed to play a key role in providing hip-extension force for propulsion and absorbing power to control knee extensions [18]. In this line, a previously injured hamstring exhibited a significant weakness in eccentric force despite players returning to full training and competition [19,20]. Thus, it is possible that soccer players with a previous HSI have an impaired sprinting performance. However, to the best of the authors’ knowledge, only one report has evaluated sprinting performance of European soccer players with a previous HSI compared with uninjured players [20]. In that study, semiprofessional soccer players returning from a recent HSI were significantly slower compared to the uninjured players, and two months of regular soccer training after returning to sports resulted in significant improvement in sprinting speed (acceleration). Nevertheless, no investigation has to date studied sprinting performance in previously hamstring-strained elite soccer players belonging to professional European teams.

Given the well-known link between a prior hamstring injury and the increased risk of suffering a subsequent injury in professional soccer [5], a greater understanding of the features displayed by previously injured players is required. We hypothesized that players with a history of a unilateral HSI exhibit eccentric knee flexor strength during the NHE that was lower in the previously injured limbs compared to both the contralateral uninjured limb and the uninjured limbs in the previously uninjured players. Therefore, the aim of the present study was to investigate whether players with a history of a unilateral HSI exhibit: (a) unilateral deficits in knee flexor eccentric strength and/or hamstring muscle volume, and (b) differences in sprinting performance.

## 2. Materials and Methods

### 2.1. Participants

The present investigation studied 46 elite professional soccer players (18.4 ± 1.6 yr; 177.5 ± 1.0 cm; 71.0 ± 6.2 kg) belonging to two of the reserve squads of two Spanish La Liga clubs during the 2017–2018 season. In the last five seasons, the first team squad of one of the clubs has been ranked among the top three while the other team has held positions ranging from 22 to 37 of the official UEFA ranking (https://www.uefa.com/nationalassociations/uefarankings/club/#/yr/2017 (accessed on 19 December 2021)). Usually, the players had ~9 h of soccer training with one or two competitive matches per week. To be authorised for participation in the investigation, players were required to be injury free at the beginning of the study. The purpose and experimental procedure were described to the players and written informed consent was obtained. The current investigation was approved by the Institutional Research Ethics Committee (i.e., Qatar Antidoping Lab—IRB number: E2013000004) following the recommendations of the Declaration of Helsinki.

### 2.2. Study Design

A retrospective cohort design was used in the present investigation. All players were tested in the first week of preseason training (July). First, all players underwent magnetic resonance imaging (MRI) measurements. On a different day, after a standardized warm-up, players carried out a 20-m running sprint test (with 5 and 10 m split times) and the Nordic hamstring exercise, while recording the eccentric knee flexor strength of the left and right limbs. MRI measurements were completed on a different day. Players were familiarized with the exercise procedures prior to the beginning of each test, and they were asked not to perform intense exercise on the day prior to the testing session. After the testing session, participants were divided into two groups: players who sustained a unilateral hamstring strain injury in the 24 months before the testing sessions and injury free players.

### 2.3. Imaging Technique and Hamstring Muscle Volume

Images were obtained in two centers on different MR scanners (Cannon MR 3T Vantage Titan and 1.5T GE Optima 360, Tokyo, Japan) with the same image protocol. T1-weighted axial MR images (Repetition time: 750 ms, Echo time: 12 ms, Field of View: 400 mm; slice thickness: 10 mm; gap: 0 mm) were obtained of both limbs from the iliac crest to the head of the fibula.

Muscle volume was calculated using the OsiriX Software (OsiriX Lite V.9.0, Geneva, Switzerland) by manual segmentation. The images were postprocessed by a blinded radiologist specialized in musculoskeletal imaging (+10 years of experience) with a Workstation Vitrea Advanced of Vital Images (Canon Medical Systems Company, Tokyo, Japan). The boundary of each muscle (long head of biceps femoris, short head of biceps femoris, semimembranosus, and semitendinosus) was drawn with 10 slices each, avoiding the connective peripheral tissue, vessels, and scars because these are tissues which do not change with physical activity. Each muscle volume was reconstructed and measured by the software with all the cross-sectional areas drawn.

### 2.4. Eccentric Knee Flexor Strength

The assessment of eccentric knee flexor strength was performed using the Nordic hamstring exercise (NHE) field-testing device (NeuroExcellence, Porto, Portugal). The testing procedures were the same as a previous study with soccer players [14], with the players knelt over a padded board with ankles secured closely superior to the lateral malleolus by individual ankles braces attached to uniaxial load cells (Sensocar, Barcelona, Spain) to measure the force [14,21]. Participants received the same instructions as in previous articles [14,21]: progressively lean forward at the slowest possible velocity resisting this movement with both limbs keeping the trunk and the hips in a neutral position and with the hands kept across the chest. Players performed a standardized warm up before testing, consisting of mobility exercises, active stretching exercises focused on the posterior chain, and three gradual repetitions of the NHE. Following this, players performed three repetitions with correct adherence to the proper technique and with 30 s recovery between repetitions. The highest peak force was recorded for further comparisons and the peak force for each of them was averaged for statistical analysis. 

### 2.5. Sprinting Performance

Sprinting performance was assessed by a 20-m sprint time with 5-m and 10-m split times. The running test was executed outdoors with appropriate weather circumstances on an artificial turf field. The front foot was placed 0.5 m before the first timing gate, and players started when ready, eliminating reaction time. Time was recorded with photoelectric cells (Microgate, Bolzano, Italy). The 20-m running sprint test was executed two times, separated by one minute of passive recovery. The best time in each one of the variables was taken for subsequent analysis.

### 2.6. Retrospectively HSI Reporting

The recruited soccer players had sustained unilateral hamstring strain injuries in the 24 months before the first testing session. The details of their history of injuries were obtained from their club doctors. Details obtained included playing position (goalkeeper, defender, midfielder, or attacker), which limb was injured (dominant/nondominant limb), type of injury (muscle disorder, muscle strain injury I, II, or III), and severity of the injury (slight/minimal [0–3 d], mild [4–7 d], moderate [8–28 d] or severe [>28 d]). MRI performed 48 to 72 h after the injuries confirmed all diagnoses.

### 2.7. Statistical Analyses

The statistical tests were made using a social sciences package (SPSS Statistics 20, SPSS Inc., Chicago, IL, USA). Descriptive statistics were calculated on each variable and the Shapiro–Wilk test was used to verify normality. Data are presented as means ± standard deviations (SD). The differences between previously injured limbs and uninjured limbs among the injured players were determined using the Student’s dependent *t*-test. The differences between previously injured limbs and all uninjured limbs among the hamstring injury-free players were determined using the Student’s independent *t*-test. All analyses were carried out with the level of significance set at *p* ≤ 0.05. In addition to the null hypothesis testing, the effect size (ES, 95% confidence interval) was determined, and the magnitude of the change in the effect size was classified as trivial (0.0–0.19), small (0.2–0.49), medium (0.5–0.79), and large (≥0.80) [22]. In addition, possible differences were analysed employing magnitude-based inferences by prespecifying 0.2 between-subject SDs as the smallest worthwhile effect. Chances of higher or lower differences were evaluated qualitatively as possibly, 25% to 74.9%; likely, 75% to 94.9%; very likely, 95% to 99.5%; and most likely, >99.5% [23].

## 3. Results

### 3.1. Players and Prior Injury Details

From the total of 42 football players, 11 sustained a total of 21 hamstring injuries during the two previous competitive seasons. There were no statistical differences in age, body mass, and height between uninjured players and players who had experienced a previous hamstring strain (Table 1). Information on the players’ characteristics and details of the injury histories in previously injured players are presented in Table 1.

### 3.2. Eccentric Knee Flexor Strength

**Figure 1:**
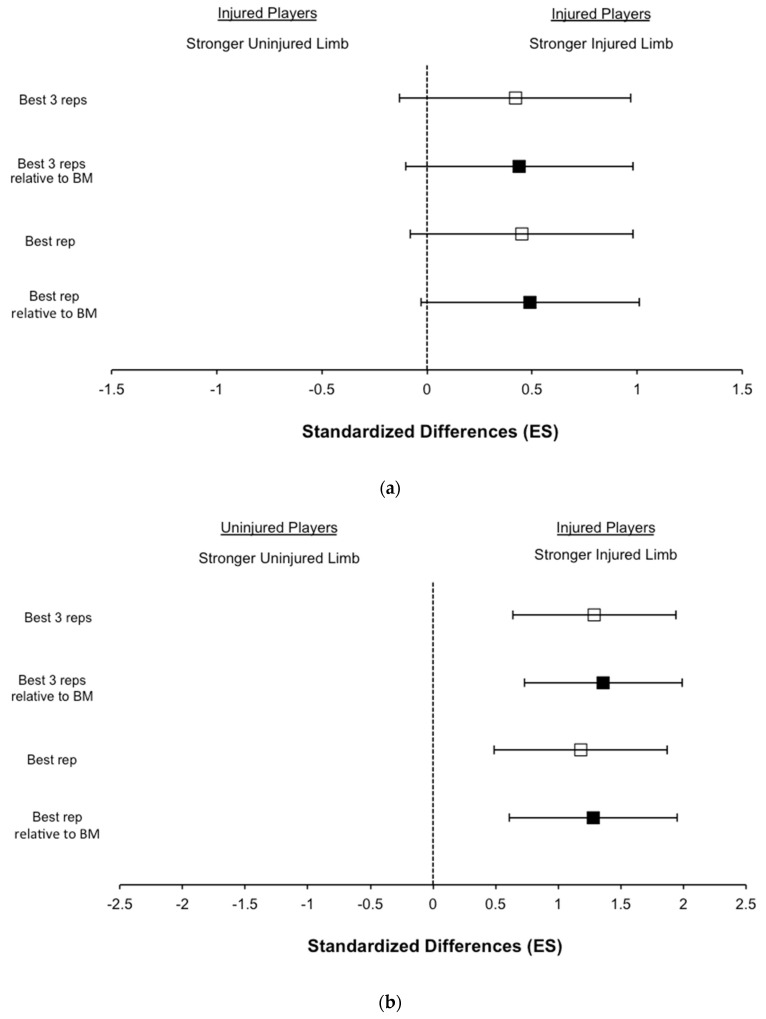
Standardized differences in knee flexor eccentric strength assessed with a Nordic hamstring exercise. (**a**) Comparisons between the injured limbs vs. uninjured limbs among players with a prior hamstring injury, (**b**) Comparisons between the injured limbs of players with a prior hamstring injury vs. all uninjured limbs among the uninjured players.

Knee flexor eccentric strength results are shown in Table 2 and Figure 1. When comparing the prior strained limb to the uninjured limb among injured players, all strength variables analysed were likely greater (small ES: 0.42–0.49, *p* = 0.061–0.064) in previously injured limbs (Figure 1 and Table 2). Similarly, when comparing the injured limbs to the limbs of uninjured players, all strength variables analysed were significantly greater (large ES: 1.18–1.36, *p* < 0.05) in previously injured limbs (Table 2).

### 3.3. Hamstring Muscle Volume

**Figure 2:**
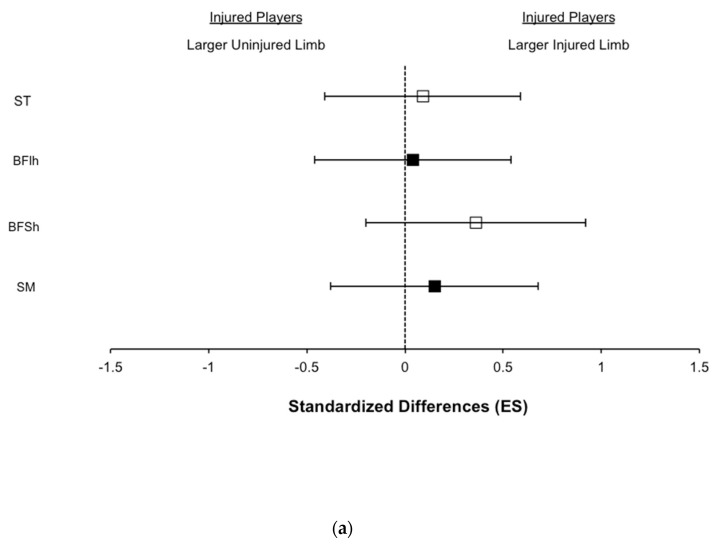

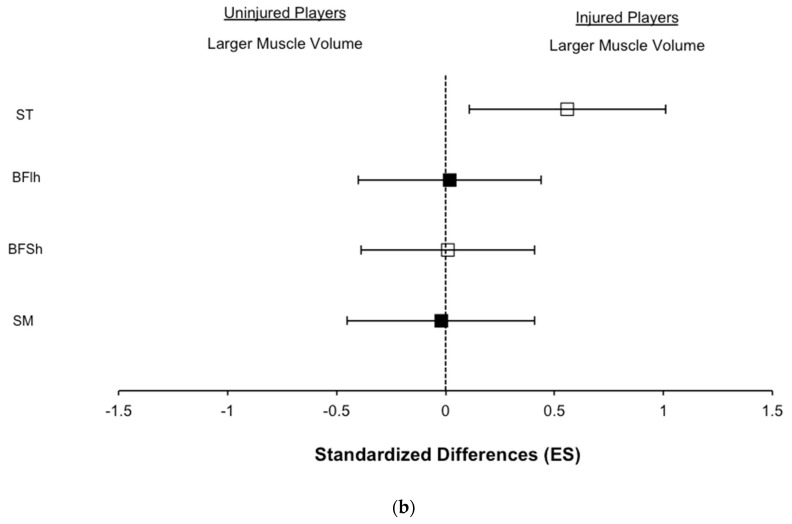
Standardized differences in hamstring muscle volume for the semitendinosus (ST), biceps femoris long head (BFlh), biceps femoris short head (BFSh), and semimembranosus (SM) muscles assessed with magnetic resonance image (MRI). (**a**) Comparison between the injured limbs vs. uninjured limbs among players with a prior hamstring injury. (**b**) Comparison between the injured limbs of players with a prior hamstring injury vs. all uninjured limbs among the uninjured players.

Hamstring muscle volume results are displayed in Table 3 and Figure 2. The previously injured limbs had possibly larger BFSh muscle volume (small ES: 0.36; *p* = 0.09) than the contralateral uninjured limbs among the injured players. The previously injured limbs likely had larger ST muscle volumes than the limbs of the uninjured players (medium ES: 0.56; *p* = 0.07).

### 3.4. Sprinting Performance

Running sprint performance results are shown in Table 4. Players who had experienced a previous HSI were possibly slower in the 5-m (small ES: −0.46; *p* = 0.1) while unclear differences were found in the 10- and 20-m times.

## 4. Discussion

Hamstring injuries remain high in football [3], which might indicate that the preventive measures currently implemented are largely ineffective. Therefore, the aim of the present study was to investigate, for the first time in the same cohort of professional soccer players, whether players with a history of HSI exhibit bilateral differences in knee flexor eccentric strength, hamstring muscle volume, and sprinting performance. The main finding was that the eccentric knee flexor strength during the NHE was greater in the previously injured limbs compared to both the contralateral uninjured limbs in the previously injured group (small ES: 0.42–0.49) and the uninjured limbs in the previously uninjured players (large ES: 1.18–1.36). Additionally, in soccer players with a history of HSI, BFSh muscle volume was possibly greater in the previously injured limbs compared to the contralateral uninjured limbs (small ES: 0.36), while ST muscle volume was greater in the previously injured limbs compared to the limbs of the uninjured players (medium ES: 0.56). Moreover, 5-m sprinting performance was possibly worse in players with a history of hamstring muscle strain injury, compared to the previously uninjured players (small ES: −0.46).

Perhaps surprisingly, potential secondary risk factors, which can be altered via a range of interventions, for recurring hamstring injuries in football players have been scarcely investigated [7,19]. Specifically, in high-level, professional football players these factors remain largely unexplored. In this regard, low eccentric knee flexor strength has been suggested as one of the main influential factors in reinjury in football players in low-level football players [6,19]. Despite its perceived importance, no previous study has specifically compared knee eccentric strength of previously injured vs. uninjured elite football players. Current results indicate that previously injured limbs displayed a possibly greater NHE strength than the uninjured contralateral limbs and greater than the uninjured limbs in the previously uninjured players. Previous work conducted with different athletic and nonathletic populations has found lower [24,25], similar [8,15], or greater [26] knee flexor eccentric strength in subjects with a history of hamstring strains. Due to the retrospective nature of the present study, it cannot be determined whether the observed knee flexor eccentric strength values were already present prior to the hamstring injury occurrence. Additionally, or alternatively, the players’ rehabilitation and postinjury follow-up programs after injury may have altered their knee flexors eccentric strength given the greater emphasis for the use of Nordic and other knee flexor eccentric strength exercises [10,16] in hamstring strain injury prevention and rehabilitation in recent times [5]. Indeed, recovery of strength performance to preinjury levels in lower level football players [27] and other recreational athletic populations [8,15] has been previously reported. Moreover, knee flexion eccentric strength has been reported to increase between 11% to 21% after short-term (6 to 10 weeks) hamstring-emphasized, eccentric-biased resistance training [16,28,29,30].

Observations of greater BFSh muscle volumes in the previously injured hamstrings (Figure 2 and Table 3) supports preceding research carried out in different athletic populations [9,15]. In addition, ST muscle volumes in the previously injured hamstrings were greater than the uninjured limbs of the previously uninjured players (medium ES: 0.56, Figure 2 and Table 3). Albeit speculative, the hypertrophy in the BFSh and the ST might be an exercise-induced adaptation in response to the use of heavy knee-dominant exercises such as the Nordic hamstring or others, which can typically be included in the rehab and individual prevention programs of players with previous hamstring injuries. Indeed, the greater hamstring eccentric strength in the injured limbs compared to the uninjured limbs observed in the present study indicates the superior ability of the injured limbs to withstand knee-dominant eccentric actions. In this regard, the only two studies to date analysing acute changes in muscle activity via functional MRI during Nordic hamstring in professional soccer players [31,32] reported substantial increases in use mainly in ST and BFSh. Moreover, in a sample of recreational athletes, those acute changes in ST and BFSh muscle use observed in the Nordic hamstring exercise matched quite well with the increases in muscle volume in ST (+ 21.2%) and BFSh (+15.3%) after 10 weeks of training with the same exercise [33], considering the augmented strength capabilities of the injured leg in a knee-dominant exercise.

Sprint acceleration is a very important component of running performance in professional soccer [34] and constitutes the primary HSI mechanism [2,35]. Running kinematics and sprint performance are influenced by neuromuscular fatigue and could also depend on the capacity to fully extend the knee while the player is sprinting, which might be the case for injury-related sprint technique, especially in the previously injured legs with a nonoptimal neuromuscular fatigability [36]. Only one study has to date compared sprinting performance between previously hamstring-injured and uninjured football players [20], showing that two months after being cleared to return to sport, sprinting performance was similar between previously injured and uninjured players. The present study found possibly worse (small magnitude) early acceleration (i.e., 5-m time) in previously injured players, while similar results with unclear differences were found in the 10-m and 20-m times (Table 4). The differences in the sprinting performance results of the current study and that of Mendiguchia et al., 2014 could be due the different populations (professional soccer players in the current study vs. semiprofessional). Finally, differences in sprinting performance could have been present prior to the hamstring injury occurrence. In addition, the players’ rehabilitation programs after injury may have altered their sprinting performance, similar to what happened with the muscle volume. In this regard, early engagement in sprinting speed factors (e.g., running technique, specific strength) and actual sprinting speed (both acceleration and peak speed) during hamstring injury rehabilitation has been suggested to improve sprint acceleration and associated sprint-related mechanical variables [37]. In addition, optimal therapy and rehabilitation during the return to play process is the first step to prevent a future hamstring injury [38,39], and apprehension of pain in previously injured players during maximum effort sprints might reduce the ability to realize peak acceleration performances in those players [40].

As a practical application, the first step in secondary hamstring muscle injury prevention is to identify potential cohort- and/or individual-specific modifiable risk factors for the design of targeted injury prevention strategies. Despite all the research, attention, and support that knee flexor eccentric strength has recently gained [6,19,25,26], current results do not seem to support the idea that in our homogenous cohort of high-level football players, knee flexor strength weakness assessed using the NHE, is likely to be the main factor to counteract potential injury prevention interventions. If present results are transferred to a “real life” scenario and the teams’ coaching and medical staffs are presented with current data, it would be questionable to prioritize the inclusion of NHE strength data in the previously injured group. Alternatively, it could also be speculated that the greater eccentric hamstring muscle strength observed can have a protective effect on those previously injured players. Further studies should be conducted to analyse the association between eccentric knee flexor strength assessed with the NHE, muscle morphology, and sprinting and to determine which of these modifiable factors play a role in subsequent hamstring strain injuries.

## 5. Conclusions

In conclusion, this study identified professional football players with a prior hamstring strain having greater NH strength, relatively hypertrophied semitendinosus and biceps femoris short head muscles, and possibly worse 5-m sprinting performance than previously uninjured players. This can have implications for the design of secondary hamstring muscle injury prevention strategies.

## Figures and Tables

**Table 1:** Characteristics and information regarding the prior hamstring injuries of the subjects participating in this study.

	Previously Injured Players (*n* = 11)	Uninjured Players (*n* = 31)	Standardized Differences (± 95%CL)	*p* Value
Age (yr)	19.4 ± 1.4	18.7 ± 2.0	0.56 ± 0.55	0.156
Body mass (kg)	71.7 ± 6.0	71.9 ± 5.9	0.03 ± 0.48	0.917
Height (cm)	178.6 ± 9.1	178.1 ± 7.4	0.04 ± 0.42	0.828
*Player Position, n (%)*				
Goalkeeper	0 (0)	2 (6)		
Defender	4 (36)	8 (26)		
Midfielder	3 (27)	13 (42)		
Attacker	4 (36)	8 (26)		
*Limb Dominance, n (%)*				
Right	7 (64)	22 (71)		
Left	4 (36)	9 (29)		
*Type of injury, n (%)*				
Functional disorder	7 (33)			
Muscle strain injury I	12 (57)			
Muscle strain injury II	2 (10)			
Muscle strain injury III	0 (0)			
*Severity of injury, n (%)*				
Slight/minimal (0–3 d)	2 (10)			
Mild (4–7 d)	4 (19)			
Moderate (8–28 d)	12 (57)			
Severe (>28 d)	3 (14)			
*Side of the Injury, n (%)*				
Dominant	13 (62)			
Non-dominant	8 (38)			

**Table 2:** Comparisons of knee flexor eccentric strength assessed with the Nordic hamstring exercise between the injured players (i.e., players with a history of a previous injury within the last two competitive seasons) and all uninjured limbs in the uninjured players.

	Previously Injured Player				Uninjured Player		
	Injured Limb	Uninjured Limb	% Differences (± 95%CL)	*p* Value	All Uninjured Limbs	% Differences (± 95%CL)	*p* Value
Best three reps (N)	330.1 ± 31.3	317.4 ± 33.8	+4.0 ± 5.1	0.077	298.1 ± 64.7	+11.8 ± 5.6	0.033
Relative to BM	4.6 ± 0.4	4.4 ± 0.4	+4.0 ± 4.8	0.061	4.2 ± 0.8	+11.8 ± 5.1	0.013
Best rep (N)	344.6 ± 28.5	331.8 ± 30.2	+3.8 ± 4.4	0.077	318.3 ± 66.5	+9.7 ± 5.3	0.083
Relative to BM	4.8 ± 0.4	4.6 ± 0.4	+3.8 ± 4.0	0.064	4.4 ± 0.8	+9.7 ± 4.8	0.001

BM, body mass.

**Table 3:** Comparison of muscle volume for the semitendinosus (ST), biceps femoris long head (BFlh), biceps femoris short head (BFSh), and semimembranosus (SM) muscles assessed with magnetic resonance image (MRI) between the injured players (i.e., players with a history of a previous injury within the last two competitive seasons) and all uninjured limbs in the uninjured players.

	Previously Injured Player				Uninjured Player		
	Injured Limb	Uninjured Limb	% Differences (95%CL)	*p* Value	All Uninjured Limbs	% Differences (95%CL)	*p* Value
ST (cm^3^)	271 ± 30	268 ± 30	+1.1 ± 5.8	0.36	255 ± 37	+6.3 ± 4.9	0.07
BFlh (cm^3^)	221 ± 41	219 ± 41	+0.8 ± 9.7	0.66	220 ± 30	+0.5 ± 8.2	0.95
BFSh (cm^3^)	141 ± 33	131 ± 31	+7.7 ± 11.4	0.09	140 ± 25	+0.3 ± 8.9	0.85
SM (cm^3^)	258 ± 36	252 ± 39	+2.2 ± 7.7	0.33	259 ± 44	−0.3 ± 6.4	0.88

**Table 4:** Comparison of sprinting performance between the injured players (i.e., players with a history of a previous injury within the last two competitive seasons) and all uninjured limbs in the uninjured players.

	Injured Players	Uninjured Players	% Differences (95%CL)	Standardized Differences (95%CL)	*p* Value
5-m sprint (s)	1.03 ± 0.05	1.01 ± 0.05	−2.1 ± 2.1	−0.46 ± 0.47	0.11
10-m sprint (s)	1.74 ± 0.07	1.72 ± 0.10	−1.3 ± 2.3	−0.34 ± 0.59	0.40
20-m sprint (s)	2.97 ± 0.09	2.97 ± 0.09	−0.2 ± 1.4	−0.08 ± 0.46	0.79

## Data Availability

The data presented in this study are available on request from the corresponding author.
